# The Portuguese Rheumatoid Arthritis Impact of Disease (RAID) score and its measurement equivalence in three countries: validation study using Rasch Models

**DOI:** 10.1007/s11136-018-1959-4

**Published:** 2018-08-01

**Authors:** Ricardo J. O. Ferreira, Laure Gossec, Cátia Duarte, Joanne K. Nicklin, Sarah Hewlett, J. A. P. da Silva, Mwidimi Ndosi

**Affiliations:** 1 Rheumatology Department, Centro Hospitalar e Universitário de Coimbra, Coimbra, Portugal; 2 Health Sciences Research Unit: Nursing (UICiSA:E), Escola Superior de Enfermagem de Coimbra, Coimbra, Portugal; 3 Sorbonne Universités, UPMC Univ. Paris 06, Institut Pierre Louis d’Epidémiologie et de Santé Publique, GRC-UPMC 08, Paris, France; 4 Rheumatology Department, AP–HP, Pitié-Salpetrière Hospital, Paris, France; 5 Coimbra Institute for Clinical and Biomedical Research (iCBR), Faculty of Medicine, University of Coimbra, Coimbra, Portugal; 6 Department of Nursing and Midwifery, University of the West of England, Bristol, UK; 7 Academic Rheumatology Unit, University Hospitals Bristol, Bristol, UK

**Keywords:** Rheumatoid arthritis, Health-related quality of life, Patient-reported outcome measures, Validation studies, Cross-Cultural comparison, Disease impact

## Abstract

**Purpose:**

The Rheumatoid Arthritis Impact of Disease (RAID) score assesses seven impact domains of interest for people with RA. This study aimed to test patients’ understanding of the Portuguese RAID and evaluate its cross-cultural validity for use in Portugal.

**Methods:**

This was a mixed methods study comprising two phases: (i) cognitive debriefing to determine patient’s comprehension of the Portuguese RAID and (ii) cross-cultural validation using Rasch analysis. Construct validity was determined by fit to the model, invariance culture (compared with France and UK datasets) and evidence of convergent and divergent validity.

**Results:**

Patients’ input (*n* = 38) led to minor changes in the phrasing of two items to ensure conceptual equivalence between the Portuguese and the original RAID. In Rasch analysis (*n* = 288), two items ‘Sleep’ and ‘Physical well-being’ in the Portuguese dataset did not adequately fit the model specifications, suggesting multidimensionality (sleep—not necessarily associated with RA) and redundancy (physical well-being overlapping with functional disability). Despite the imperfections, the scale had high internal consistency, evidence of convergent and divergent validity and invariance to culture (compared to France *n* = 195 and UK *n* = 205 datasets). The scale was well targeted for patients with different levels of disease impact.

**Conclusions:**

The RAID has been successfully adapted into Portuguese and it can be used with confidence in clinical practice. Further research will be required to ensure it captures the full range of sleep problems in RA. Meanwhile, data across the three countries (Portugal, France and the UK) are comparable except for the two items (sleep and physical well-being).

**Electronic supplementary material:**

The online version of this article (10.1007/s11136-018-1959-4) contains supplementary material, which is available to authorized users.

## Introduction

Rheumatoid arthritis (RA) is the most common and serious inflammatory arthritis [1]. The clinical presentation of RA is not circumscribed to joint inflammation, its main characteristic, but having a systemic involvement. Its symptoms such as pain, fatigue, morning joint stiffness, sleep disturbances, or depression highly affect patient’s quality of life [2, 3]. Because of this, the patient’s perceptions of the impact of the disease are of upmost importance.

Different patient-reported outcomes (PROs) have been developed to assess the patient’s perspective and are included as endpoint for clinical trials [4, 5]. The “Rheumatoid Arthritis Impact of Disease” (RAID) score is one of these PROs. It was developed in English, through a European League Against Rheumatism (EULAR) initiative, aiming at combining the most important domains for patients in one single measure [6, 7]. Several methodological steps were followed and are detailed elsewhere [6, 7]. Briefly, the first step was to identify important health domains affected by RA through a focus group with ten patients from ten European countries. These domains were then ranked by 96 patients based on their personal experiences and the seven highest-ranked domains were selected (pain, functional disability assessment, fatigue, sleep, physical well-being, emotional well-being and coping with disease). The wording and the scaling of the domains resulted in seven numerical rating scales (NRS), from 0 (best status) to 10 (worst status). The relative weights for each domain were then defined based on patient priorities (pain weighs 21%, functional disability 16%, fatigue 15%, and the remaining 12%). Finally, a longitudinal observational study of 570 patients from 12 European countries revealed its construct validity, reliability and sensitivity to change [6, 7]. Later studies supported these psychometric properties and acceptability to patients, who also commented on its ease of completion [8–10].

The RAID score has now been used in clinical trials [11], registries [8] and also in national audits [12], both as a global score and for individualized goal setting in clinical practice, using its seven items individually instead of its global weighted score [13–16]. This assumes validity and reliability of the global score and of each item of RAID, although the latter had not been established until now. The number of translations of the RAID (over 70 languages) [17] also demonstrates the interest in its use. A Portuguese translation for Portugal and for Brazil was performed by a specialized company (Mapi Institute) in 2012 [17]. However, the Portuguese RAID required further input from patients in Portugal and robust psychometric evaluation of the individual items as well as the global score in order to ensure valid measurements at the individual and group levels. In addition, the evidence of measurement comparability (psychometric equivalence) between the Portuguese RAID and the original RAID score needed to be demonstrated in order to allow data-pooling and comparison across countries. Modern psychometric methods such as Rasch analysis allow for detailed evaluation of psychometric properties of a scale, thus validating the adapted version, as well as testing measurement comparability across scales [18]. The aim of this study was therefore to test patients’ understanding of the Portuguese RAID and evaluate its cross-cultural validity using Rasch analysis.

## Participants and methods

### Study design

This was a mixed methods study comprising two phases: (1) cognitive debriefing [19] with patients to assess face validity of the existing Portuguese version of RAID and (2) cross-cultural validation of the Portuguese RAID to assess its construct validity, reliability and cross-cultural invariance.

### Participants

Consecutive adults with a definite diagnosis of RA [20, 21] attending a rheumatology outpatient department in Portugal were included. Patients were excluded only if they were unable to respond to the questionnaires unaided.

In parallel, anonymized datasets of patients with RA from the UK and France [22] were used to test the RAID’s cross-cultural invariance.

### Data collection

In Phase 1 (Cognitive debriefing), Portuguese patients with diverse demographic and clinical characteristics were invited to individual interviews or focus groups (with 3–4 patients). The previously translated Portuguese RAID was used; this score is freely available and not copyrighted [17]. Patients were firstly asked to complete the Portuguese RAID unaided; then they were asked to assess possible difficulties and to probe what they thought was meant by each domain with questions such as “*What do you think this item means? What did you think or consider before giving a number*/*answer to it?*”. All interviews were conducted, audio-recorded and transcribed *verbatim* by same researcher (RF).

In Phase 2 (validation study), a consecutive sample of patients completed a questionnaire that included the RAID, demographic data (gender, age, educational background), self-reported disease duration and other PROs to enable testing the RAID’s convergent and divergent validity: Health Assessment Questionnaire Disability Index (HAQ-DI) [23], Functional Assessment of Chronic Illness Therapy-Fatigue (FACIT) [24], patient global assessment (PGA) of disease activity (0–100 mm visual analogue scale (VAS)) [25], Hospital Anxiety and Depression Scale (HADS) [26], quality of life (EQ-5D) [27], Subjective Happiness Scale (SHS) [28], and Ten-Item Personality Inventory (TIPI) [29]. Physician global assessment (PhGA) of disease activity (0–100 mm VAS) and treatment were retrieved from medical records. Patients were considered as experienced on NRS if they had responded to a scale with NRS format in rheumatology more than once in the last year, otherwise, they were considered not experienced.

### Data analysis

Qualitative data from phase 1 were analysed using inductive and realistic thematic analysis according to Braun and Clarke [30] guidelines and managed using ATLAS.ti 8, Berlin: Scientific Software Development GmbH. Two authors (RF, CD) independently analysed all the transcripts. Team discussions showed that they reached similar conclusions.

In phase 2 of this study, Rasch analysis was used to test whether the Portuguese RAID had retained its psychometric properties following the adaptation process. The Rasch model provides a mathematical framework against which data from questionnaires can be formally assessed to determine how they conform to propreties of fundamental measurement [31]. Fit to the model implies construct validity, reliability, unidimensionality and statistical sufficiency of the total score from the scale. Consequently, interval estimates can be derived from the scale for use in parametric analyses [32, 33].

The raw scores of each of the seven RAID items were used in all analyses, not the weighted scores. For Rasch analysis, RUMM2030 software (Perth, WA: RUMM Laboratory Pty Ltd) was used with Master’s Partial Credit Model parameterization [34]. The software uses pairwise conditional maximum likelihood for estimating item parameters. Model fit is tested by Chi-square-based fit statistics comparing the difference between observed responses and the expected values using standardized residuals (null hypothesis being no significant difference between the observed and the values expected by the model). Fit to the model is supported by (i) item-person interaction statistics, expressed as a Z score with a mean of zero (range − 2.5 to 2.5) and standard deviation (SD) of one and (ii) non-significant Chi-square probability. Additionally, an important aspect of measurement, the invariance of the tool to different levels of the construct being measured, is tested by (iii) item-trait Chi-square interaction statistic with a non-significant probability indicating the invariance of the tool, in this case, the invariance of the RAID to different levels of RA impact. A more detailed description of the Rasch analysis approach, its use in rheumatology and the interpretation of fit statistics is given elsewhere [31] .

In addition to the fit statistics, three further tests were conducted within the framework of Rasch analysis, to assess the properties of the RAID. These are threshold ordering, unidimensionality and invariance of the scale to culture. Each RAID item has 11 response categories, which reflect an ordered continuum of higher impact of the domain (from 0 to 10). The ‘threshold’ is the point between two adjacent categories, where either response is equally probable [35]. When data fit the Rasch model, thresholds should be correctly ordered and this is tested statistically and presented graphically. Disordered thresholds mean that participants have difficulty to consistently discriminate between response categories [31]. Although fit to the model implies that the scale is unidimensional, the principal component analysis and *t* test-based method, first proposed by Smith [36], were used as a unidimesionality confirmatory test. For this test, first two sets of items hypothesized to represent low levels and high levels of the trait/construct (disease impact) are defined based on the correlation between items and the first residual factor, then an independent *t* test is used to compare the difference in these estimates for each person. Unidimensionality is confirmed if ≤ 5% of the *t* tests are significant or if lower bound of a binomial 95% CI of the observed proportion overlap 5% [36]. Differential item functioning (DIF) analysis within RUMM2030 software is based on a two-way analysis of variance (ANOVA) of residuals across each level of person factor (age group, gender, education background, disease duration, culture) and across different levels of trait (disease impact). The presence of uniform DIF is suggested if the *p* value of the main effects (person factor e.g. age, culture) is significant. If the *p* value of the interaction effect (person factor X level of trait) is significant, this suggests a non-uniform DIF [37]. The absence of DIF by country implies cross-cultural invariance [22].

To assess the convergent and divergent validity, Pearson’s correlation coefficients between the RAID items and the other measures were calculated and interpreted as good, |*r* ≥ 0.60|; moderate, |*r* = 0.40–0.59| and poor |r < 0.40| correlation [38]. The hypotheses tested were that individual items of the RAID score would have (i) good correlation with corresponding measures of physical function, fatigue and psychological distress (i.e. RAID items 2 and 5 with HAQ-DI, RAID item 3 with FACIT, and RAID items 6 with HADS) as well as good correlations of all RAID items and global measures such as health-related quality of life (EQ-5D) and patient global assessment (PGA) of disease activity; and (ii) poor correlation with measures of unrelated constructs such as happiness (SHS), personality traits (TIPI) and physician global assessment (PhGA).

A *p* value of 0.05 was considered significant except where a Bonferroni adjustment was applied to account for multiple testing i.e. 0.05/number of tests. Missing data were not imputed. Analyses were conducted using IBM® SPSS® Version 20.0. Armonk, NY: IBM Corp. and RUMM2030 software, Perth, WA: RUMM Laboratory Pty Ltd.

## Results

### Patient characteristics

In total, 38 patients were included in Phase 1 and 288 in Phase 2. Their demographic and clinical characteristics are summarized in Table 1. Most patients were women, with low education background and with long standing disease duration.

**Table 1:** Characteristics of patients included in the Portuguese dataset

Variable	Phase 1 (*n* = 38)	Phase 2 (*n* = 288)
Gender (female) [*n* (%)]	32 (84.2)	236 (81.9)
Age [years, mean (SD)]	58.6 (10.4)	59.8 (12.4)
Education background [*n* (%)]		
≤ 4 years	22 (57.9)	139 (48.3)
5–9 years	4 (10.5)	66 (22.9)
10–12 years	7 (18.4)	49 (17.0)
> 12 years	5 (13.2)	34 (11.8)
Disease duration [years, mean (SD)]	15.7 (11.3)	11.8 (8.9)
Treated with bDAMRDS [*n* (%)]	25 (65.8)	87 (30.2)
DAS28(CRP)4v [mean (SD)]	na	2.9 (1.0)
Experienced with NRS [*n* (%)]	26 (68.4)	149 (51.7)
HAQ-DI remission (≤ 0.5) [*n* (%)]^a^	na	77 (27.3)
PGA (0–100) [mean (SD)]	na	44.5 (26.8)

*bDMARD* biological disease-modifying antirheumatic drugs, *DAS28(CRP)4v* Disease activity assessment using 28-joint counts, *C*-reactive protein and four variables, *HAQ-DI* Health Assessment Questionnaire Disability Index, *na* not assessed, *NRS* Numeric Rating Scale, *PGA* Patient Global Assessment, *SD* standard deviation

^a^Missing data in 6 (2.1%) patients

### Phase 1: cognitive debriefing

Few issues were raised about understanding of some terminologies used in four items (items 3, 4, 5 and 6); however, only two items (item 3 and item 4) were subject to minor changes:

Item 3—“Fatigue. Circle the number that best describes how much fatigue you felt due to your RA during the last week”. Some patients did not know the meaning of “Fadiga” (Fatigue); the term “Cansaço” (tiredness) was known by everyone:

“I understand what fatigue is but tiredness I would have understood better.” (Patient 7)

After discussion, it was agreed to add the second term to the head of the item—“Fadiga/Cansaço”.

Item 4—“Sleep: circle the number that best describes the sleep difficulties (i.e. resting at night) you felt due to your RA during the last week”. In the Portuguese version the term “difficulties” was translated into “distúrbios” (disturbances). For some patients this term was difficult to understand, as it may be understood as “disease/diagnose” or as “difficulty to sleep” and the problem could be “sleep too much”:

“Sometimes I sleep bad because of other problems, related to me, not related to the pain, thank God. (...) Maybe it would be better to use «loss of sleep» or other simpler word [instead of disturbances]...” (Patient 5) “Maybe «changes», although disturbances is understandable...” (Patient 7), “or «difficulties»...” (Patient 6)

It was agreed to change the term “distúrbios” to “dificuldades” (difficulties).

Item 5—“Physical well-being: Considering your arthritis overall, how would you rate your level of physical well-being during the past week?”. For most patients to be in a good state of physical well-being (“Bem-estar físico”) was described as: not having pain, followed in terms of frequency by being able to walk, and in lesser extent, being able to perform domestic/work tasks and few patients referred to other meanings. Among patients, the concept meaning appeared to overlap with item 2 (functional disability assessment/“Avaliação de deficiência funcional”), which assesses the difficulty in doing daily physical activities. The researchers considered this as a conceptual problem rather than a translation issue. Supplementary Table S1 presents patient’s quotes related to the four items.

Beyond the terminologies used in the four items, the anchors of the NRS were also discussed because at least two patients assumed that ten correspond to better health condition, disregarding what was written in the NRS’s anchors. One patient made an analogy with the grades of a school’s test:

“Yes, it is like the grades: 5 is nothing but 10 is already a good mark. Because of that people may think that 10 is good.” (Patient 15).

Since this was a major change, without enough support, no changes were done to these anchors.

### Phase 2: cross-cultural validation

#### FIT to the model

Table 2 presents item fit statistics for each country and for the pooled data. Table 3 presents item-person fit statistics, person separation reliability and unidimensionality of the RAID. Two items (sleep and physical well-being) had fit residual values outside the acceptable (− 2.5 to 2.5) range; Sleep fit residual = 4.218, *p* = 0.007, suggesting that this item may be measuring a slightly different dimension and Physical well-being fit residual = − 4.239, *p* = 0.014, suggesting redundancy of this item (corroborates the findings of our qualitative study—patient interpretation of physical well-being overlapped with functional disability). To test the extent to which lack of fit was caused by this redundancy an attempt was made to combine the Functional disability and Physical well-being items into a testlet to address the local dependence (Table 2b). This resulted in better fit statistics for both items (Functional disability/Physical well-being fit residuals − 3.680, *p* = 0.373, and sleep fit residual 3.552, *p* = 0.069). The item-trait Chi-square probabilities in each country data were also non-significant but the item fit residual for Portugal dataset remained higher (SD = 2.303) than expected (SD = 1) (Table 3b). This suggests that local dependency was not the only cause for lack of fit.

**Table 2:** Item fit statistics for each country dataset and the pooled data

Country	RAID Item	Location	SE	Fit residual^a^	DF	X^2^	*p* value^b^
A: Fit statistics for the original 7-item structure							
Portugal (*n* = 288)	Pain	− 0.072	0.045	0.622	5	2.506	0.776
	Functional disability	− 0.162	0.044	− 1.464	5	2.300	0.806
	Fatigue	− 0.188	0.043	0.211	5	2.638	0.756
	Sleep	0.265	0.041	**4.218**	5	15.840	**0.007**
	Physical WB	− 0.202	0.046	− 4.239	5	14.213	0.014
	Emotional WB	0.058	0.043	− 0.120	5	2.074	0.839
	Coping	0.300	0.043	− 0.154	5	0.931	0.968
UK (*n* = 205)	Pain	− 0.018	0.049	− 0.456	3	2.245	0.523
	Functional disability	0.041	0.048	− 0.848	3	1.936	0.586
	Fatigue	− 0.358	0.049	1.986	3	6.374	0.095
	Sleep	0.074	0.042	2.298	3	2.753	0.431
	Physical WB	− 0.138	0.051	− 2.316	3	5.325	0.149
	Emotional WB	0.044	0.047	1.483	3	6.003	0.111
	Coping	0.354	0.049	− 0.583	3	2.295	0.513
France (*n* = 195)	Pain	0.071	0.051	0.126	3	0.387	0.943
	Functional disability	0.312	0.051	− 1.856	3	5.280	0.152
	Fatigue	− 0.348	0.048	− 0.053	3	1.529	0.676
	Sleep	0.123	0.045	**4.030**	3	11.879	0.008
	Physical WB	− 0.306	0.052	− 1.452	3	3.850	0.278
	Emotional WB	− 0.058	0.049	1.280	3	1.032	0.793
	Coping	0.206	0.051	− 0.741	3	1.731	0.630
Pooled (*n* = 688)	Pain	− 0.022	0.028	− 0.341	9	4.393	0.8837
	Functional disability	− 0.003	0.027	− 2.747	9	11.875	0.2205
	Fatigue	− 0.274	0.027	1.121	9	9.124	0.4259
	Sleep	0.161	0.024	**6.000**	9	26.725	**0.0016**
	Physical WB	− 0.193	0.028	− 4.912	9	27.081	**0.0014**
	Emotional WB	0.032	0.027	1.520	9	10.735	0.2943
	Coping	0.300	0.027	− 1.079	9	4.943	0.8392
Expected values for perfect fit				Within ± 2.5			> 0.0071
B: Fit statistics after combining functional disability and physical well-being—6-item structure							
Portugal (*n* = 288)	Pain	− 0.091	0.043	0.101	233.670	2.779	0.734
	Funct. Dis./Phys.WB	− 0.204	0.030	− 3.680	233.670	5.365	0.373
	Fatigue	− 0.205	0.042	− 0.263	233.670	2.902	0.715
	Sleep	0.222	0.039	**3.552**	233.670	10.222	0.069
	–						
	Emotional WB	0.027	0.042	− 0.536	233.670	2.768	0.736
	Coping	0.251	0.041	− 0.559	233.670	1.432	0.921
UK (*n* = 205)	Pain	− 0.024	0.048	− 0.675	162.83	2.529	0.470
	Funct. dis./phys. WB	− 0.058	0.033	− 2.770	162.83	1.130	0.770
	Fatigue	− 0.341	0.048	1.713	162.83	5.853	0.119
	Sleep	0.064	0.041	1.928	162.83	1.384	0.709
	–						
	Emotional WB	0.031	0.046	1.196	162.83	5.467	0.141
	Coping	0.328	0.047	− 0.764	162.83	2.766	0.429
France (*n* = 195)	Pain	0.077	0.050	0.003	153.67	0.410	0.938
	Funct. dis./phys. WB	− 0.016	0.035	− 4.556	153.67	6.770	0.080
	Fatigue	− 0.327	0.047	− 0.186	153.67	1.412	0.703
	Sleep	0.127	0.044	**3.742**	153.67	9.773	0.021
	–						
	Emotional WB	− 0.055	0.048	1.144	153.67	0.837	0.840
	Coping	0.194	0.050	− 0.822	153.67	1.878	0.598
Pooled (*n* = 688)	Pain	− 0.033	0.027	− 0.812	557.83	5.114	0.824
	Funct. dis./phys.WB	− 0.117	0.019	− **6.655**	557.83	17.664	0.039
	Fatigue	− 0.272	0.026	0.634	557.83	9.237	0.416
	Sleep	0.14	0.024	**5.249**	557.83	18.287	0.032
	–						
	Emotional WB	0.014	0.026	1.005	557.83	9.678	0.377
	Coping	0.268	0.026	− 1.456	557.83	6.057	0.734
Expected values for perfect fit				Within ± 2.5			> 0.0083

*DF* Degree of Freedom, *WB* Well-being, *RAID* Rheumatoid Arthritis Disease Impact

^a^Large positive values suggest multidimensionality and large negative values suggests local response dependence (item redundancy)

^b^Bonferroni adjusted *p* value for model fit (i.e. 0.05/7 tests = 0.0071 and 005/6 tests = 0.0083); Figures in bold represent values bellow adjusted *p* value threshold, indicating misfit of the item to the model

**Table 3:** Summary item-person fit statistics for each country-specific and the pooled datasets

Country	Item fit residual		Person fit residual		Item-trait–Chi-square interaction		Person separation reliability (PSI)^b^	Proportion of independent *t* tests (binomial 95% CI)^c^
	Mean	SD	Mean	SD	Value (DF)	*p* value^a^		
A: Item-person fit statistics of the scale in original structure (7 items)								
Portugal (*n* = 288)	− 0.132	2.525	− 0.674	1.601	40.501 (35)	0.240	0.940	0.070 (0.045–0.095)
UK (*n* = 205)	0.223	1.718	− 0.441	1.372	40.501 (35)	0.173	0.932	0.085 (0.055–0.115)
France (*n* = 195)	0.191	1.989	− 0.712	1.571	25.690 (21)	0.219	0.914	0.090 (0.059–0.121)
Pooled (*n* = 688)	− 0.063	3.476	− 0.652	1.551	94.876 (63)	0.006	0.932	0.072 (0.056–0.089)
B: Item-person fit statistics of the scale with combined Functional Disability and Physical well-being (6-items)								
Portugal (*n* = 288)	− 0.231	2.303	− 0.660	1.484	25.468 (30)	0.702	0.934	0.063 (0.038–0.088)
UK (*n* = 205)	0.105	1.829	− 0.440	1.277	19.130 (18)	0.384	0.927	0.060 (0.030–0.090)
France (*n* = 195)	− 0.113	2.709	− 0.672	1.431	21.080 (18)	0.275	0.910	0.074 (0.043–0.105)
Pooled (*n* = 688)	− 0.339	3.882	− 0.632	1.435	66.037 (54)	0.126	0.928	0.067 (0.050–0.083)
Expected values for perfect fit	0	1	0	1		> 0.05	> 0.85^b^	Lower bound CI < 0.05

*CI* confidence interval, *DF* degrees of freedom, *SD* standard deviation

^a^ *p* value > 0.05 for model fit

^b^*PSI* Person separation index (internal consistence) value > 0.70 is acceptable for group use and > 0.85 for individual use

^c^Unidimensionality is supported if ≤ 5% (0.05) of independent *t* tests are significant or if lower-bound of a binomial 95% CI of the observed proportion overlap 5% (0.05)

#### Threshold ordering and targeting

**Fig. 1:**
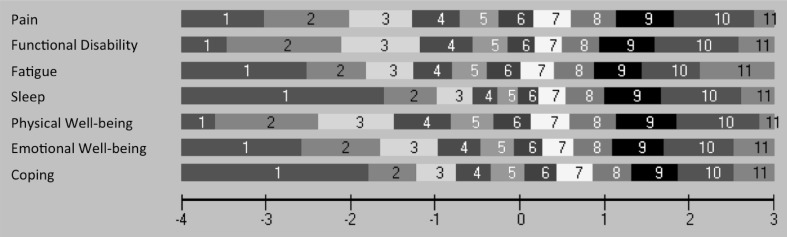
Threshold map for Portuguese RAID. The items are in ranked order of difficulty according to Rasch model (*n* = 288). RAID items were answered in a numerical rating scale from 0 to 10, values represented here as response categories from 1 to 11. It would be expected that as the person’s disease impact increases, they would be more likely to obtain a higher score and that this would increase systematically in a logical progression

**Fig. 2:**
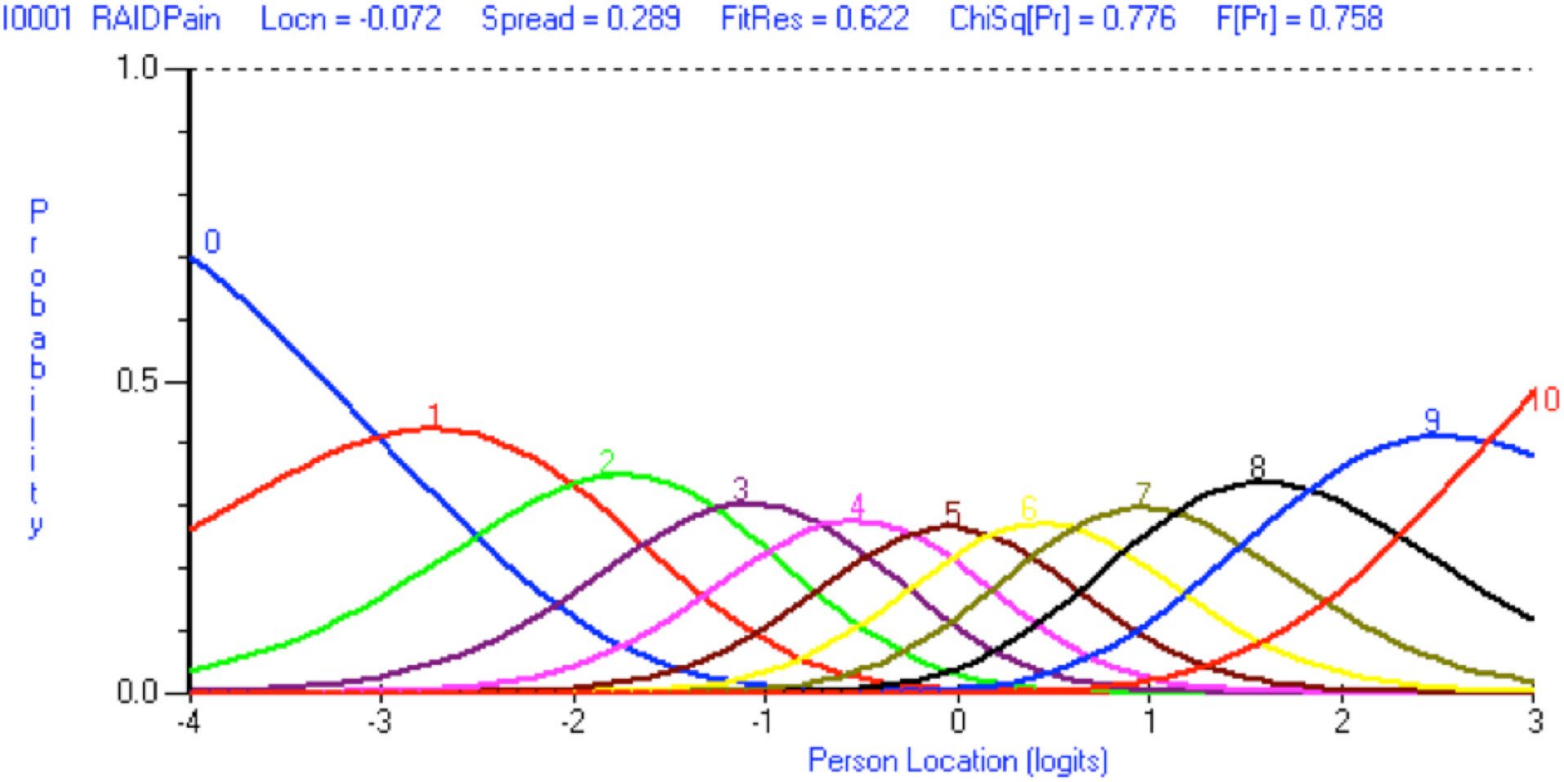
Category probability curve for item 1 (Pain) of Portuguese RAID (*n* = 288). The *x-*axis represents the construct, with the pain increasing to the right. The *y*-axis shows the probability of scoring the categories: Each of the 11 categories emerged as the most likely to be selected at some point upon the underlying pain scale, showing logical progressive order

**Fig. 3:**
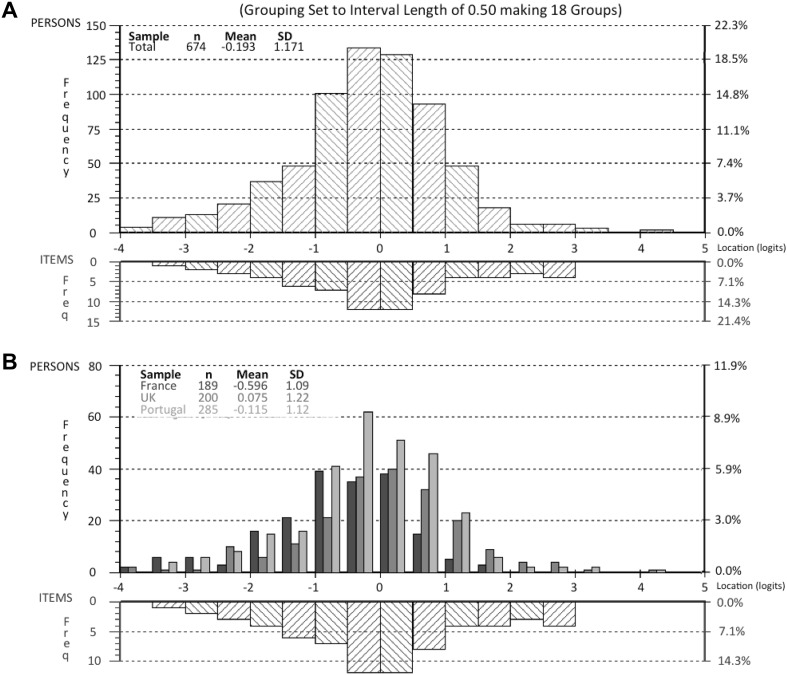
Person-item threshold distribution for the 7 items of the Rheumatoid Arthritis Impact of Disease (RAID) score. These graphs present the distribution of items: the x-axis is the logit score and represents the interval scaling of the items according to the Rasch model, with − 4 being no impact and 5 being high impact of disease. The lower part of each histogram is where individual items are located along the scale; the top part of histogram represents the number of people and their total RAID logit score. **a** Represents the pooled analysis of all patients, showing very good fit between person and items. **b**. Represents the persons divided by the three countries, also with very good fit. French patients have more impact of the disease as the central part of the distributions goes more into the left than the other two countries. In both diagrams the extremes (*n* = 14) were omitted

The thresholds for all seven items were ordered, suggesting that the proposed NRS response structure was working as intended (Fig. 1). This is also shown in the category probability curves (Fig. 2). The scale was well targeted for patients with different severity levels of RA impact (Fig. 3).

#### Unidimensionality and internal consistency

The reliability of Portuguese RAID was excellent (PSI = 0.940) and the strict unidimensionality test revealed the proportion of significant *t* tests to be 0.070 (95% CI 0.045–0.095), supporting the unidimensionality of the scale (Table 3).

#### Invariance

There was no DIF by country on any of the RAID items; therefore, cross-cultural validity of the RAID is supported (Table 4). Furthermore, the scale was invariant to age, gender, education background, disease duration and functional capacity.

**Table 4:** Cross-cultural DIF of the RAID items by age and by country

Dataset	RAID domain	Age^a^			Country		
		Mean square	F (DF)	*p* value^b^	Mean square	F (DF)	*p* value^b^
Portugal (*n* = 288)	Pain	0.05	0.06 (1)	0.81	Not applicable		
	Functional disability	0.37	0.49 (1)	0.48			
	Fatigue	0.01	0.01 (1)	0.93			
	Sleep	0.45	0.34 (1)	0.56			
	Physical WB	0.13	0.24 (1)	0.62			
	Emotional WB	1.90	2.29 (1)	0.13			
	Coping	2.11	2.55 (1)	0.11			
UK (*n* = 205)	Pain	0.50	0.62 (1)	0.43	Not applicable		
	Functional disability	0.63	0.81 (1)	0.37			
	Fatigue	1.43	1.32 (1)	0.25			
	Sleep	0.14	0.12 (1)	0.73			
	Physical WB	0.12	0.19 (1)	0.66			
	Emotional WB	0.19	0.19 (1)	0.66			
	Coping	3.78	4.85 (1)	0.03			
France (*n* = 195)	Pain	1.30	1.52 (1)	0.22	Not applicable		
	Functional disability	3.32	5.53 (1)	0.02			
	Fatigue	1.40	1.64 (1)	0.20			
	Sleep	0.00	0.00 (1)	0.96			
	Physical WB	0.02	0.03 (1)	0.87			
	Emotional WB	4.70	4.93 (1)	0.03			
	Coping	0.21	0.28 (1)	0.60			
Pooled (*n* = 688)	Pain	0.07	0.09 (1)	0.770	0.90	1.07 (2)	0.343
	Functional disability	4.70	6.66 (1)	0.010	2.90	4.21 (2)	0.015
	Fatigue	1.64	1.80 (1)	0.180	3.85	4.22 (2)	0.015
	Sleep	2.26	1.72 (1)	0.190	1.25	0.98 (2)	0.376
	Physical WB	0.02	0.03 (1)	0.966	1.19	0.32 (2)	0.727
	Emotional WB	3.15	3.37 (1)	0.067	0.21	0.23 (2)	0.797
	Coping	8.35	10.55 (1)	0.001	1.11	1.41 (2)	0.246

*WB* Well-Being

^a^Categorized as < 60 years and ≥ 60 years old

^b^Bonferroni adjusted *p* value for model fit: 0.05/7 tests = 0.0071

#### Convergent validity

As hypothesized, the correlation of RAID items with other PROs (Table 5) was good (*r* ≥ 0.60), namely between HAQ-DI and “RAID Functional disability” and “RAID Physical well-being” (*r* = 0.66 and *r* = 0.67, respectively), FACIT and “RAID Fatigue” (*r* = − 0.65) [also good correlations with “RAID Physical well-being” and “RAID emotional well-being”, *r* = − 0.60 for both], HADS depression and “RAID emotional well-being” (*r* = 0.61), and between PGA and between EQ-5D with all RAID items, except with “RAID sleep” which had moderate correlations (*r* = 0.57 and *r* = − 0.52, respectively). The remaining correlations between RAID items and the other PROs were moderate (0.40 ≤ *r* < 0.60), as expected.

**Table 5:** Pearson’s correlations between the RAID (transformed scores) and other outcomes in the Portuguese dataset (*n* = 288) to demonstrate criterion-related validity

Other measures	Pain	Functional disability	Fatigue	Sleep	Physical WB	Emotional WB	Coping
With expected good correlations (convergent validity)							
HAQ-DI	0.56^a^ (< 0.001)	0.66^b^ (< 0.001)	0.61^b^ (< 0.001)	0.58^a^ (< 0.001)	0.67^b^ (< 0.001)	0.59^a^ (< 0.001)	0.61^b^ (< 0.001)
FACIT	− 0.50^a^ (< 0.001)	− 0.51^a^ (< 0.001)	− 0.65^b^ (< 0.001)	− 0.55^a^ (< 0.001)	− 0.60^b^ (< 0.001)	− 0.60^b^ (< 0.001)	− 0.58^a^ (< 0.001)
HADS—anxiety	0.42^a^ (< 0.001)	0.45^a^ (< 0.001)	0.51^a^ (< 0.001)	0.49^a^ (< 0.001)	0.51^a^ (< 0.001)	0.54^a^ (< 0.001)	0.50^a^ (< 0.001)
HADS—depression	0.47^a^ (< 0.001)	0.53^a^ (< 0.001)	0.54^a^ (< 0.001)	0.50^a^ (< 0.001)	0.55^a^ (< 0.001)	0.61^b^ (< 0.001)	0.59^a^(< 0.001)
EQ-5D	− 0.61^b^ (< 0.001)	− 0.69^b^ (< 0.001)	− 0.66^b^ (< 0.001)	− 0.52^a^ (< 0.001)	− 0.71^b^ (< 0.001)	− 0.64^b^ (< 0.001)	− 0.62^b^ (< 0.001)
PGA	0.65^b^ (< 0.001)	0.69^b^ (< 0.001)	0.66^b^ (< 0.001)	0.57^a^ (< 0.001)	0.70^b^ (< 0.001)	0.64^b^ (< 0.001)	0.69^b^ (< 0.001)
Without expected good correlations (divergent validity)							
PhGA	0.18 (0.002)	0.17 (0.004)	0.13 (0.029)	0.11 (0.051)	-0.04 (0.527)	0.16 (0.007)	0.12 (0.042)
SHS	− 0.26 (< 0.001)	− 0.24 (< 0.001)	− 0.32 (< 0.001)	− 0.30 (< 0.001)	− 0.31 (< 0.001)	− 0.38 (< 0.001)	− 0.37 (< 0.001)
TIPI—extraversion	− 0.21 (0.001)	− 0.23 (< 0.001)	− 0.24 (< 0.001)	− 0.23 (< 0.001)	− 0.22 (< 0.001)	− 0.22 (< 0.001)	− 0.22 (< 0.001)
TIPI—agreeableness	0.00 (0.978)	− 0.02 (0.724)	− 0.51 (0.396)	− 0.05 (0.428)	− 0.04 (0.527)	− 0.09 (0.120)	− 0.11 (0.070)
TIPI—conscientiousness	− 0.00 (0.910)	− 0.09 (0.125)	− 0.09 (0.129)	− 0.11 (0.070)	− 0.09 (0.144)	− 0.14 (0.020)	− 0.11 (0.065)
TIPI—emotional stability	− 0.21 (< 0.001)	− 0.27 (< 0.001)	− 0.26(< 0.001)	− 0.25 (< 0.001)	− 0.32 (< 0.001)	− 0.32 (< 0.001)	− 0.26 (< 0.001)
TIPI—openness to experiences	− 0.11 (0.064)	− 0.14 (0.017)	− 0.20 (0.001)	− 0.09 (0.144)	− 0.20 (0.001)	− 0.17 (0.004)	− 0.21 (< 0.001)

Values represent: *r* (*p* value)

*EQ-5D* EuroQol-5D, *FACIT* Functional Assessment of Chronic Illness Therapy-Fatigue, *HADS* Hospital Anxiety and Depression Scale, *HAQ-DI* Health Assessment Questionnaire Disability Index, *PGA* Patient Global Assessment, *PhGA* Physician Global Assessment, *RAID* Rheumatoid Arthritis Disease Impact, *SHS* Subjective Happiness Scale, *TIPI* Ten Item Personality Inventory, *WB* Well-Being

^a^Correlations were categorized as moderate if 0.40 ≤ *r* < 0.60 [38]

^b^Correlations were categorized as good if *r* ≥ 0.60 [38]

#### Divergent validity

The correlations of RAID items with PhGA, with happiness (SHS) and with Personality traits (TIPI) were, as expected, poor (*r* < 0.40) or not statistically significant (Table 5).

## Discussion

This study aimed to incorporate patient perspective in the adaptation of the Portuguese RAID, an impact questionnaire for RA, and to validate it for use in Portugal using modern statistical approaches.

The Portuguese version of the RAID, as well as many of its translations to other languages, was carried out by Mapi Institute, an organization specialized in PROs research. However, the Portuguese RAID required further input from a wider group of patients before the statistical evaluation of the underlying construct was carried out [19]. Our qualitative study (phase I) added more patients’ perspective into the Portuguese RAID, making it more meaningful to them. As a result, minor changes were performed in Fatigue and Sleep items to ensure that patients understand the concepts intended by the items and thus give accurate responses.

The cognitive debriefing also revealed that some patients may not read the item anchors which might led them to interpret 10 as good when it means bad. This misunderstanding has also been found in other studies regarding VAS interpretation by patients with chronic conditions [39–41]. This problem might have been resolved by adding smiley faces at the extremes like in pain assessment for children or elderly persons, but it would have been a major change to the tool that is already established. Providing a brief explanation about the anchors of the scale may therefore be important especially for first time users or for those patients with low literacy or low educational level [39, 42].

The results of Rasch analysis highlighted two issues (i) redundancy of Physical well-being (overlapping with functional disability) and (ii) the possibility that Sleep item measures a slightly different dimension. These issues were not observed in the UK dataset (the original RAID was developed in English) suggesting that the problem may not be inherent to RAID itself but reflecting imperfections of the cross-cultural adaptations. Addressing the redundancy by combining the two locally dependent items (physical well-being and functional disability) improved the scale performance, including fit residuals for the sleep item. Future developments need to address the phrasing of physical well-being to avoid confusion with functional disability. As sleep item still displayed higher SD than expected by Rasch model, the improvement (seen by combining functional disability and physical well-being) was not sufficient enough to justify changing the RAID structure into a 6-item questionnaire, although this presented a better fit to the model. Dimensionality of the sleep item needs further exploration. Sleep problems include difficulties in initiating sleep, maintaining sleep, early awakening and non-restorative sleep [43]. Given the broad range of sleep problems, it is possible that some patients do not necessarily associate them with RA impact: “*Sometimes I sleep bad because of other problems”, a patient said*. A qualitative study of the Dutch RAID found that sleep was hardly mentioned in the focus groups with patients [44]. It may be helpful to investigate whether conceptual equivalence issues exist in the Portuguese translation and/or adding explanation to the item to help patients associate sleep problems with RA impact.

Despite lack of adequate fit to the Rasch model, construct validity of the Portuguese RAID is supported by (i) high internal consistency (PSI > 0.85) suggesting enough reliability for use at the individual level (ii) good targeting for patients with different levels of disease impact (iii) good correlations (convergent validity) between RAID items and other tools measuring the same concepts i.e. HAQ-DI, FACIT, HADS, EQ-5D, PGA; and (iv) poor correlations (divergent validity) between RAID items and tools measuring different concepts i.e. PhGA, happiness and personality traits. The first study to test RAID performance in clinical practice (*N* = 1086) was conducted in Norway,[8] and used weighted RAID global score and showed good correlation with PGA, Rheumatoid Arthritis Disease Activity Index (RADAI), Short-Form (SF)-36 general health, SF-6D and EQ-5D (*r*_p_ was 0.82, 0.82, − 0.66, − 0.77 and − 0.73, respectively) [8]. In our study of convergent validity looking at individual RAID items, only the Sleep item showed a moderate correlation with global measures of health (PGA *r*_p_ = 57, and EQ5D *r*_p_ = − 0.52), the rest of items had good correlations. Indeed, in the above-mentioned Norwegian study [8] the weighted RAID global score also had a moderate correlation (*r*_p_ = 0.59) with the Sleep Medical Outcome Study (MOS) subscale. It should be noted that specific sleep measures have been shown to have higher correlations with the individual RAID item measuring sleep (*r*_p_ = 0.73–0.78) [45]. Our study did not use specific sleep measures hence the modest correlations.

This study has three limitations. First, lack of adequate fit to Rasch model observed in the Sleep and Physical Well-being items in the Portugal datasets meant that we could not calibrate a stable interval scale from our data (statistical sufficiency of the total score and interval-level scaling depends on adequate fit to the model) [31]. New datasets will therefore need to be tested for fit if an interval-level scale is desired. The lack of fit may be related to either un-resolved conceptual equivalence issues in the translation (explained above) or the restrictive nature of Rasch model. Rasch model specifies that all items contributing to the scale have uniform discrimination—i.e. all items have parallel item characteristic curves. The model detects even minor variations in items discrimination across different levels of RA impact. It would be useful in future studies to assess the performance the RAID using other item response models (2-parameter logistic or 3-parameter logistic models) which can accommodate a degree of variation in item discrimination [46]. However, fit to these alternative models imply less precise interval scale estimates than those derived from Rasch model. It may as well be useful to explore how well the RAID works as an ordinal measure of disease impact using non-parametric item response models such as Mokken scaling [47]. Second, the Portuguese dataset was compared with only two countries, France and the UK. While our data have demonstrated comparability of measures across the three countries, the evidence supporting cross-cultural equivalence would have been stronger if fit to Rasch model was adequate. We therefore downgrade our conclusions to apply to the fitting items only (excluding physical well-being and sleep). Further research will be required to assess measurement equivalence of the Portuguese RAID and other adapted versions data are compared. Lastly, it is important to note that all our analyses were based on unweighted RAID scores as completed by patients. The RAID scoring involves applying different weights to different items to obtain clinically meaningful measures [6, 7].

The strengths of this study include the following: the recruitment of enough Portuguese patients in both phases, with good diversity, including patients who are not used to completing NRS. The use of Rasch model in our analysis enabled a robust assessment of psychometric properties of the RAID including different aspects of construct validity and response bias, although this may also mean a highly strict criteria for measurement validity above that required for clinical use. Availability of datasets from the UK and France allowed assessment psychometric equivalence between the Portuguese RAID, the original RAID and the French version.

Beyond research, this study has important clinical implications. As RAID is a valid measure of the impact of disease in RA, without which many PROs would be required, this study provides Portuguese patients with a measure that is meaningful to them. It will help clinicians and other health professionals assess how RA impacts on patients. Also, the RAID has been incorporated into the Portuguese National Registry [48] which will help inform the impact of RA at the population level and provide data for further research.

In conclusion, cross-cultural adaptation and validation of the RAID score into Portuguese for Portugal has been achieved using cognitive debriefing and Rasch models. Although further research will be required to address issues observed in the Sleep and Physical well-being items, the Portuguese RAID has enough construct validity and reliability for clinical use at individual patient and group level.

## Electronic supplementary material

Below is the link to the electronic supplementary material.

Supplementary material 1 (DOCX 25 KB)
