# Supplementary material for: The Portuguese Rheumatoid Arthritis Impact of Disease (RAID) score and its measurement equivalence in three countries: validation study using Rasch Models
Source: Qual Life Res. 2018 Aug 1;27(11):2909–21. doi: 10.1007/s11136-018-1959-4 (PMC6208571; doi:10.1007/s11136-018-1959-4)
Supplement: Supplementary file 1 — Supplementary material 1 (DOCX 25 KB) [file 11136_2018_1959_MOESM1_ESM.docx]

**Supplementary Table S1.** Main quotations from cognitive debriefing

| **Item 3 - Fatigue**  *- “I understand what fatigue is but tiredness I would understood better.*” (Patient 7)  *- “Yes. Tiredness is a lot easier!”* (Patient 13)  - "*It is better to say tiredness because older people may have more difficulty with fatigue*" (Patient 14) |
| --- |
| **Item 4 - Sleep**  - "*Sometimes I sleep bad because of other problems, related with me, not related with the pain, thank God. (...) Maybe it would be better to use «loss of sleep» or other simpler word [instead of disturbances]...*" (Patient 5)  - "*Maybe «changes», although disturbances is understandable.*.." (Patient 7), "*or «dificulties».*.." (Patient 6), - "*Yes, maybe we understand [disturbances], but for others might be not so easy*." (Patient 5)  - "*These sleep distrbances are not very well because my sleep disturbance may be not sleep loss, but sleeping too much. I wondered if these disturbances are related with taking some pills... I did not find this was very explicit. People may also take it as a «disease»*." (Patient 18) |
| **Item 5 - Physical Well-being**  - "*(...) What I think about physical well-being is usually getting myself up and not having pain when I put my feet on the floor, it's being able to grab something and when I make some movement do not hurt my hands..."* (Patient 13)  - "*If I can run, if I can or not go up and down stairs as I want..."* (Patient 12)  *- “It's walking a little bit well. (...) It's no pain* [laughs]" (Patient 16)  - "*It's hold things..."* (Patient 15)  - "*Exactly. Hold things. Open a bottle. Stand."* (Patient 13) |
| **Item 6 - Emotional Well-being**  - "[emotional well-being] *It's how I feel psychologically, if I'm okay with myself, if I'm okay with life (...) Emotional and psychological ... I do not know if they are the same thing but at least they are part of a set.*" (Patient 10)  - "*People rural areas (...) when they want to speak in the psychological, speak in «thoughts», in ... «I give well with the thoughts»*"(Patient 13)  - "*Being well out of your head!* [laughs]. *Yes, is «I'm not good of my head. I do not know anymore what I say neither what I do»*".(Patient 15)  - "We *sometimes want to pick it up something and when we come to the place we do not know what it's going to fetch anymore*." (Patient 16)  **Comment**: Some discussion arose about possible differences between "emotional" ("emocionais") and "psychological" ("psicológico") problems, also if memory or attention problems could be considered in answering this item. However, it was agreed that "emocionais" is a good representation of the concept. |
| **Item Anchors**  - "*I gave a 7 [to pain] because I'm not totally good*." (Patient 1). Interviewer: "*Ah, ok. But 10 it's not good, it's bad*." "*Ohhh then I did everything the other way around ... I missed everything, I failed the test* (...) *the further I walked* [in the scale] *the more high* [grade] *I had"* (Patient 1). Interviewer: "*But did not you see what was written on the extremes of the scale?*". "No, I thought *«*until 10 I do not arrive, but until 7 ... *»*" (Patient 1).  - "(...) *some people think like that, exactly: 10 is good, he is on the state 10; It's 5 stars; It's 10 stars*." (Patient 13)  - "*Yes, it is like the grades: 5 is nothing but 10 is already a good mark. Because of that people may think that 10 is good*." (Patient 15) |
